# The Influence of Hospital Sunday

**Published:** 1910-06-18

**Authors:** 


### The Influence of Hospital Sunday.

In these islands sectarianism is more pronounced than even on the Continent, and the number of sects between which the British people divides its religious allegiance is very great. Moreover, the amount of rancour and animosity displayed between one Church and another is unfortunately still very considerable. When all this is admitted—intelligent Mahomedans travelling amongst us do not allow us long to forget it—it is satisfactory to know that amid all our schisms and disunions there is one subject upon which the rivalry between parish church, synagogue, chapel, meeting-house, and the rest is purely friendly. The occasion for this very satisfactory suspension of religious hostility is the setting apart of the day known as Hospital Sunday, whereon all creeds are united in that form of alms and well-doing which has been especially enjoined upon the Church of Christ, the care of the sick. On this one day all are content to work for one common aim, because all are aware that in the hospitals of London there is no religious test applied. The sole inquiry that is made concerns the patient's condition and his means—Is he in need of treatment? Is he unable to procure it for himself? And it is because this character is so jealously preserved in our hospitals that it is possible to appeal successfully to ministers and congregations of all types to co-operate in this universal method of helping the voluntary hospitals to carry on their work.

To the thoughtful, therefore, Hospital Sunday is an omen full of hope and encouragement for the future. Good in itself and in its immediate results, it also affords evidence that a common platform can be found upon which the most hostile and jarring theological interests do not disdain to meet. If through the institution of Hospital Sunday something has been done, and is being done, to break down those barriers of mutual distrust and suspicion which separate Christian and Jew, Catholic and Protestant, Churchman and Dissenter, then indeed have the hospitals indirectly done something that nothing else has been able to accomplish. For ignorance breeds distrust, and mutual ignorance is at the bottom of half the bitterness which exists between some of the sects. By taking an interest in the Sunday Fund, its purpose, its distribution, its effect on embarrassed hospital finances, the different Churches are necessarily drawn together in such a way that the mists of prejudice are swept away; to participate with one another in a work of mercy is one of the surest ways of laying the foundations of mutual respect and affection. There is little doubt this process has commenced, and the asperities of rival beliefs have been materially softened.

Upon the individual worshipper the beneficent influence of his opportunity is even more apparent. Once again it is ignorance that accounts for apathy, and when the Londoner is properly aroused by a well-informed preacher to the sense of what he owes the hospitals, and how he can assist in acknowledging the debt, he can generally be depended upon to rise to the occasion. He fails to support the hospitals (of the majority, at least, this is true), not because he does not like them or approve of them, but just because their pressing needs are not brought home to him with sufficient energy and clearness. With many also, no doubt, insufficient means is the reason why a regular subscription to a hospital is not part of their annual expenditure. To this last class Hospital Sunday is a great boon; for it enables those who would like to subscribe regularly, but cannot do so, to contribute according to their means and to help forward the work with what they can spare. That such contributions from humble folk are both a privilege to them, and in the aggregate a source of great advantage to the hospitals cannot be contested; and surely something of the spirit of the cheerful giver is bound to suffuse itself into the hearts of those who give for the cause of the sick and suffering.

To the preachers who plead the cause of the hospitals upon Hospital Sunday the occasion is also one of real benefit. Some, to speak plainly, are more effective than others because they have got up their facts more carefully, or because they are more eloquent or more earnest. But all, or almost all, must feel that the occasion is one which draws forth the very best that is in them; and there are many preachers on whom the gift of tongues never descends so fully as on Hospital Sunday. That the ministers of religion can and do work so magnificently year by year for this Fund is one of those things that are in danger of being taken for granted; the authorities of the hospitals are ever mindful of the splendid services thus rendered to them in the past, with a very grateful appreciation of all their magnificent work in this direction, and a confident hope that it may long continue. When the Hospital Sunday Fund was started, it is safe to say that no one contemplated the dimensions to which it would rise. And most assuredly no one could imagine or predict how profoundly it was to affect the givers; for, as we have tried to show, it has effects far wider and more far-reaching than the mere mitigation of the financial difficulties under which the London hospitals labour. The result of this year's collection is not yet known, but it is sincerely to be hoped that this Fund, in which the late King took so great an interest, will maintain its splendid record.

